# Enhancing quality of ruminant feed through fungal treatment: Usage of bamboo shoot residues

**DOI:** 10.1371/journal.pone.0302185

**Published:** 2024-05-28

**Authors:** Chunyan Huo, Yuhong Guo, Yihe Zhao

**Affiliations:** Center for Efficient Utilization of Tufted Bamboo Resources, and Center for Quality Inspection and Testing Center of Economic Forest Products, National Forestry and Grassland Administration, Yunnan Academy of Forestry and Grassland, Kunming, China; University of Agriculture Faisalabad, PAKISTAN

## Abstract

In this investigation, we explore the harnessing of bamboo shoot residues (BSR) as a viable source for ruminant feed through fungal treatment, with the overarching objective of elevating feed quality and optimizing bamboo shoot utilization. The white-rot fungi (*Wr*.*fungi*), *Aspergillus niger* (*A*.*niger*), and its co-cultures (*A*.*niger&Wr*.*fungi*) were employed to ferment BSR. And the impact of different fermentation methods and culture time on the chemical composition (Crude protein Ash, neutral detergent fibre and acid detergent fibers), enzyme activity (Cellulase, Laccase, Filter paperase and Lignin peroxidase activities), and rumen digestibility *in vitro* were assessed. The findings reveal a nota ble 30.39% increase in crude protein in fermented BSR, accompanied by respective decreases of 13.02% and 17.31% in acid detergent fiber and neutral detergent fibre content. Enzyme activities experienced augmentation post-fermentation with *A*.*niger&Wr*.*fungi*. Specifically, the peak Cellulase, Laccase, and Lignin peroxidase activities for BSR with *Wr*.*fungi* treatment reached 748.4 U/g, 156.92 U/g, and 291.61 U/g, respectively, on the sixth day of fermentation. Concurrently, NH_3_-N concentration exhibited an upward trend with prolonged fermentation time. Total volatile fatty acids registered a decline, and the Acetate/Propionate ratio reached its nadir after 6 days of fermentation under the *A*.*niger&Wr*.*fungi* treatment. These outcomes furnish a theoretical foundation for the development of ruminant feeds treated via fungal co-culture.

## 1. Introduction

Bamboo shoots, cherished culinary delights in tropical and subtropical regions, stand as a focal point on dining tables worldwide. Notably, China leads global bamboo shoot production, surpassing 5 million tons annually [1]. The intricate process of bamboo shoot sampling and processing generates copious by-products, predominantly bamboo shoot residues (BSR), encompassing bamboo shells and shoots, constituting a substantial 70% of the overall output [2]. The elevated water content in BSR poses challenges in storage and transportation, resulting in diminished utilization rates and environmental repercussions, including resource wastage and pollution [3]. Urgency looms over the imperative need for efficient BSR utilization.

The BSR has high nutritional value. Liu et al. [4] showed that the nutritional value of bamboo shoot residues was similar to or only slightly lower than that of bamboo shoots, and they were rich in cellulose, protein, fat and minerals. Previous studies have confirmed that BSR contain a variety of bioactive ingredients, such as phenolic acids, flavonoids and polysaccharides [5,6]. Despite these intrinsic values, the efficient extraction methods remain elusive, hindered by low utilization rates and prohibitive costs. Consequently, the exigency for innovative strategies to address BSR challenges becomes increasingly apparent. Now, against the backdrop of escalating competition for grain resources between humans and livestock in animal production [7], there arises a renewed focus on exploring non-cereal feedstuffs.

Although the cellulose content of BSR is high and the cell wall is of a three-dimensional structure, which hinders the utilization and degradation of rumen microorganisms [8]. Therefore, the removal of lignocellulose from plant cell walls is a key factor in improving efficient nutrient utilization by rumen microorganisms. The most used methods are ammoniation and silage [3,9]. However, they have the disadvantage of higher cost and longer time of cycle.

Fungi emerge as promising agents capable of degrading neutral detergent fiber (NDF) and acid detergent fibers (ADF), thereby fostering cellulose utilization by rumen microorganisms and augmenting rumen digestibility [10]. Fungi, especially white-rot fungi, secrete a variety of lignin decomposing enzymes and cellulose decomposing enzymes leading to the loss of cellulose, and different microorganisms have different ability to degrade cellulose [11]. The fungi of *Aspergillus niger* is generally considered as a safe (GRAS) microorganism with the ability to synthesize proteases, amylases, fiber-degrading enzymes (cellulases, hemicellulases, pectinases), lipases, and tannases [12]. Previous studies have reported the use of fungi for fermentation of tea residue [13], stalk [14] and Rapeseed Meal [15] as ruminant feeds. However, there have been rare studies on the use of different fungal fermented BSR as feed for ruminants. In addition, a mixed fermentation is commonly used in the processing of some products. Some studies show that mixed fermentation can make up for the deficiency of a single fermentation [16].

In this study, we used white-rot fungi (*Wr*. *fungi*, strain ndm 3–2), *Aspergillus niger* (*A*.*niger*, strain cgmcc3.4309) and co-culture of both (*A*.*niger*&*Wr*.*fungi*) were utilized to ferment BSR for ruminant feed production. The effects of fermented strains and duration of fermentation on the physicochemical properties, enzyme activity, and *in vitro* rumen fermentation of ruminant feeds were investigated. It laid a theoretical foundation for the production of ruminant feed by fungal fermentation of BSR.

## 2. Materials and methods

### 2.1 Fermented strains preparation

Two fermented strains including white-rot fungi (*Wr*.*fungi*) and *Aspergillus niger* (*A*.*niger*) used in the present study were purchased from Beijing Biological Conservation Center (Beijing, China). The microbial solution was stored at -20°C. Before the experiment, the fungous solution was placed in potato dextrose broth (PDB) (Aobox, Beijing, China) and activated at 28°C for 24 h.

### 2.2 Substrate preparation

The bamboo shoot residue (BSR) was the processed residue of *Dendrocalamus brandisii (Munro) Kurz*. Obtained from Yunnan, China. The fresh BSR were air-dried, then crushed and sifted through a 40-mesh sieve. The BSR is separated into glass bottles, and water was added for an approximate moisture content of 650 g/kg. Each glass bottle contains 30g of BSR powder and 55.7ml of water. Then the mixture was sterilized at 118°C for 60 min [17]. The sterilized substrate was then cooled to room temperature within the super clean workbench (BBS-DDC, Biobase, Jinan, China).

### 2.3 Cultivation

In the super clean workbench, 2.57 ml of 1*10^7^ spores/mL conidium suspension fully activated *Wr*. *fungi*, *A*. *niger* and mixed strains of *A*.*niger* and *Wr*.*fungi* were inoculated into the sterilized substrate respectively and cultured at 28°C. The number of fungi was obtained by plate counting. Each treatment was sampled on days 0, 3, 6, 9, 12, 15, and 18 of fermentation. Uninoculated BSR served as the control. A portion of the sample underwent drying at 65°C for 48 hours for subsequent chemical analysis and in vitro rumen fermentation culture, while another portion was directly employed for enzyme activity analysis.

### 2.4 Morphological assessments

#### 2.4.1 Growth curves

Optical density was measured at 600 nm by a UV–Vis spectrophotometer (UV-2600, Japan) as Yang et al. [18] described. Uninoculated medium served as control and culture for 36 h and take samples every 3 hours.

#### 2.4.2 The media growth on potato dextrose agar

In accordance with Shao et al. [19], a circular disk with a 10 mm diameter was excised from the actively growing colony edge on a Potato Dextrose Agar (PDA) plate. The mycelial disk was then inoculated at the center of a new PDA plate and incubated for 6 days at 28°C, after which the colony radius was measured.

#### 2.4.3 Scanning electron microscopy (SEM)

Fiber morphology changes of BSR on days 0, 9, and 18 of fermentation were evaluated using SEM. Simply put, the fermentation dry matter (DM) was attached to the sample table with a black tape, and its form was observed with a Hitachi S520 scanning electron microscope (Hitachi, Tokyo, Japan) magnified x600 times.

### 2.5 Chemical analysis

DM content of the samples was determined after drying samples to a constant weight at 65 ºC. Crude protein (CP) content was measured by 8400 kjeldahl analyzer (FOSS, Denmark). The ash content was measured after samples were subjected to 600°C for 6 h in a muffle furnace (Guangming Medical Instrument Co., Ltd. Beijing, China). The content of ash-free neutral detergent fiber (NDF) and ash-free acid detergent fiber (ADF) were obtained according to the methods of Van Soest et al. [20].

### 2.6 Enzyme activity assays

Lignin peroxidase (Lip) activity was estimated through the oxidation of veratryl alcohol to verataldehyde in presence of H_2_O_2_ [21]. Laccase activity was estimated by using ABTS (2,2’-azinobis(3-ethylbenzthiazoline-6-sulphonate)) as substrate [22]. Filter paperase (FPase) was estimated based on dinitrosalicylic acid (DNSA) method [23]. Activity of cellulase was measured by respective activity detection kit (Solarbio Science & Technology Co., Ltd, Beijing China).

### 2.7 *In vitro* incubation

According to the method of Xue Li et al. [11], an *in vitro* rumen fermentation was used to evaluate the efficacy of the fungal treatment, with some modifications. In simple terms, rumen fluid was collected in the morning from three six-month-old goats fed silage and bamboo shoots (ratio of 70:30, DM basis) for 2 wk before the trial. Subsequently, 30ml of rumen fluid and 0.6g of the sample were introduced into glass bottles and cultivated in a shaker (Tyst Instrument Co., Ltd., Tianjin, China) at 39°C and 80 rpm for 72 hours. The fermentation products were centrifuged at 10000g for 15 min and the volatile fatty acids (VFA) in the supernatant were determined by gas chromatography [24], Ammonia-N (NH_3_-N) concentration was determined by spectrophotometry using phenol hypochlorite method [25]. The pH value of the supernatant was measured using a PHSJ-3F Laboratory pH Meter (Shanghai, China).

### 2.8 Statistical analysis

The experiment was repeated in twice and the data were subjected to a one-way analysis of variance (ANOVA) for each experiment to determine significant differences among mean values using Tuckey tests with SPSS.26 (Chicago, USA). Significance was declared when the P-value was less than 0.05. In addition, mean values of each individual sample were analyzed as a completely randomized design using the general linear model (GLM) procedure of SPSS.26, while data of chemical composition and the loss of each component were subjected to two-way analysis of variance with the fixed main effects of treatment (T), fermentation days (D) and the interaction between treatment and fermentation days (T*D) using GLM procedure of SPSS.26.

## 3. Results and discussion

### 3.1 Microbial co-cultivation dynamics

#### 3.1.1 Microbial growth curves

**Fig 1:**
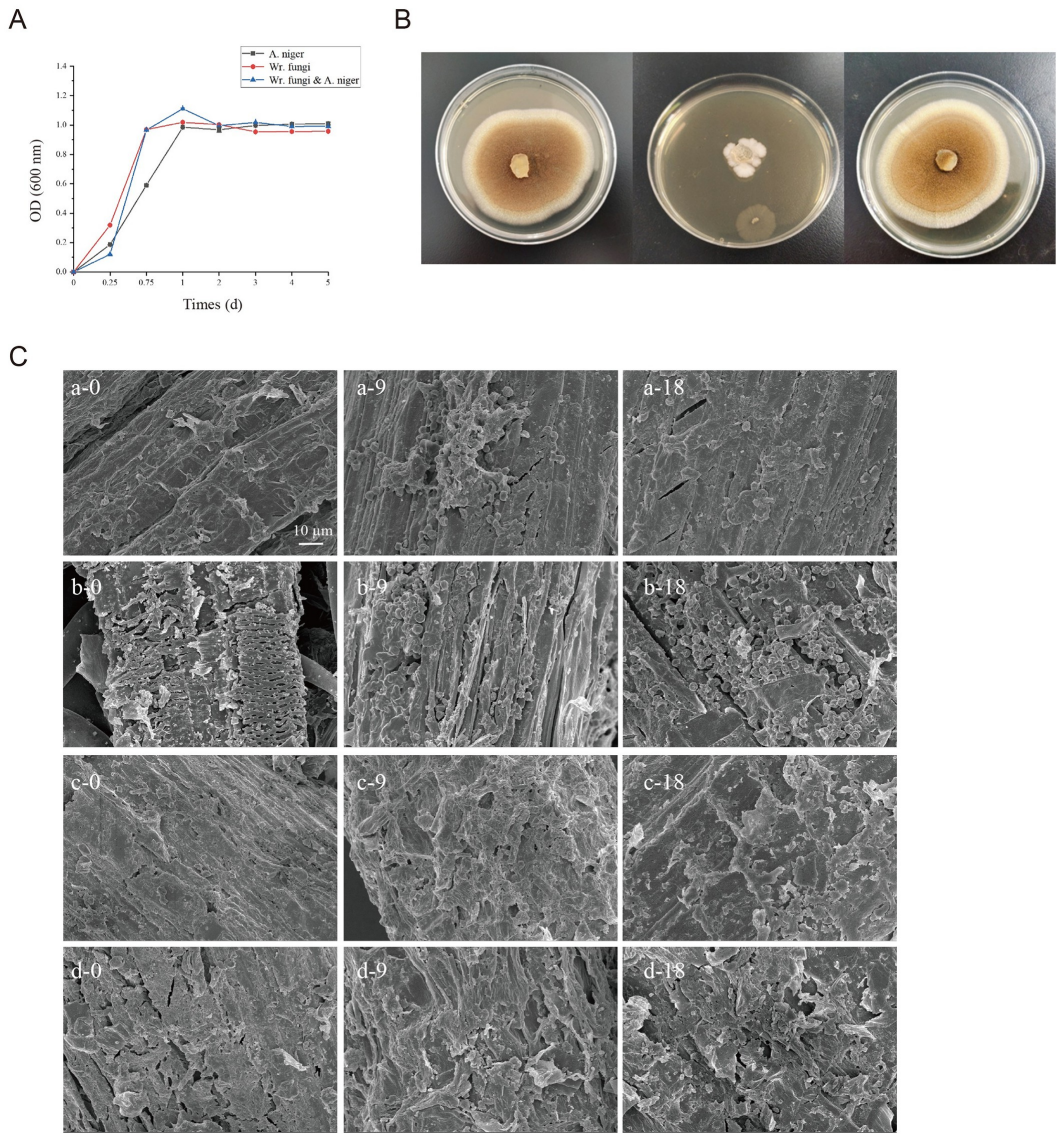
Growth curves (A); Microbial growth on PDA (B): *A*. *niger*(left), *Wr*. *Fungi* (middle) *and A*.*niger*&*Wr*.*fungi* (right); Scanning electron microscopy (SEM) of BSR fermented for 0, 9, 18 days (C): Control (a), *A*.*niger*&*Wr*.*fungi* (b), *Wr*. *fungi* (c), *A*. *niger* (d).

The optimal timing of inoculation and interaction of microorganisms can be assessed by the growth curve [18]. The growth curve of *Wr*.*fungi*, *A*.*niger* and mixed culture of strains of *A*.*niger*&*Wr*.*fungi* can be observed in Fig 1A. Our data unveil a congruent growth trend among all microbial entities, characterized by a brief lag period (0–0.25 d), followed by logarithmic growth phases spanning 0.25–1 d and 1–5 d, ultimately leading to a stable period. Notably, the optical density (OD) peaked after a one-day cultivation period. The presence of N-acetylglucosamine, recognized as a pivotal growth-promoting nutrient associated with specific strains and species [26], showcased comparable trends in our research. Intriguingly, co-culture exhibited heightened growth compared to individual cultures, potentially attributed to augmented N-acetylglucosamine production through mixed fermentation. Notably, findings from Yang et al. [18] corroborate our observations, emphasizing the superior growth curve of mixed fermentation over singular counterparts.

#### 3.1.2 The microbial growth on potato dextrose agar

The growth of different microorganisms on the potato dextrose agar (PDA) is shown in Fig 1B. Remarkably, the colony radius of *A*.*niger&Wr*.*fungi* (7.00±0.61) surpassed that of *A*.*niger* (6.80±0.44) and *Wr*.*fungi* (1.73±0.19). Both the growth curve (Fig 1A) and colony radius (Fig 1B) analyses affirm the absence of antagonism between *A*.*niger* and *Wr*.*fungi*, laying a foundational premise for co-culture fermentation in BSR animal feed production. Discrepancies in colony radius among treatments may stem from variations in spore size and environmental influences [27]. The darker hue of A.niger, attributed to drier PDA conditions compared to PDB, aligns with previous research indicating that spores with higher pigment content fare better in arid environments (Fig 1B) [28].

#### 3.1.3 Microstructure of fermented bamboo shoots remains

The morphological changes of cellulose during fermentation can be directly demonstrated by scanning electron microscopy. In this study, scanning electron microscopy was used to observe the structure of the fibers at 0, 9, and 18 days after four different treatments (Fig 1C). In the control group, the microtube bundle was tightly arranged so the microtube wall was smooth. The structure was complete and dense, and there was a large amount of lignin (a-0). This phenomenon persisted after 18 days of treatment (a-18). Following microbial fermentation, a pronounced loosening of fiber structure, gradual blurring of fiber boundaries, and increased visibility with prolonged treatment time were observed (b-18, c-18, d-18). These changes signify lignocellulose degradation by microorganisms, concurrently facilitating microbial attachment and carbohydrate absorption [11]. Moreover, co-culture fermentation introduced a profusion of spores onto exposed fibers, inducing damage to the cellulose structure, akin to observations in the study by Niu et al. [10].

### 3.2 Changes in chemical composition

Fermented strains and duration of fermentation have important effects on the change of chemical composition [11].

The content of CP after fermentation was significantly higher than that of control group (P<0.05), And it increased with the increase of fermentation time. The ability of fungi to increase protein content was as follows: *A*.*niger*&*Wr*.*fungi > A*.*niger > Wr*.*fungi*. Notably, a 30.39% increase in CP content was achieved after 12 days of *A*.*niger&Wr*.*fungi* fermentation, reaching 16.19%.

The surge in protein content post-fermentation is attributed to the breakdown of cellulose by microorganisms, releasing detectable and absorbable protein molecules, aligning with previous findings [10,11]. The ash content of the fermentation products treated with A.*niger* and *A*.*niger*&*Wr*.*fungi* increased significantly with fermentation time (P<0.05), A study by Cui yiyan et al. [13] found similar conclusions, This is because *A*.*niger* can adsorb metal ions in water, resulting in increased Ash content [29].

The content of ADF and NDF is an important index to evaluate feed quality of ruminants [20]. NDF includes cellulose, hemicellulose, and lignin as the major components, which includes the cross linked matrix of the plant cell wall and, as coarse fiber, forms the rumen mat that stimulates rumen function [30]. Acid detergent fiber is intended as a preparation for the determination of cellulose, lignin, ADIN, acid-insoluble ash (AIA), and silica. It is not a valid fiber fraction for nutritional use or for the prediction of digestibility [20].

In this study, the contents of ADF and NDF decreased significantly with the extension of fermentation time (P<0.05). And the contents of ADF and NDF after fungal treatment were lower than those of control group. These results indicate that fungi can degrade lignocellulose in BSR, which is difficult for ruminants to use, so as to promote ruminants’ digestion of cellulose and absorption and utilization of other nutrients [14]. In addition, the lowest ADF content of *Wr*.*fungi* fermented BSR was 36.69%, which decreased by 13.02%. The NDF content of BSR fermented with *A*.*niger*&*Wr*.*fungi* was the lowest, which was 60.58% at the end of fermentation, decreased by 17.31%. Fungi can secrete enzymes to degrade lignocellulose. Although fungal fermentation can degrade ADF and NDF, the utilization of cellulose was selective [31].

**Fig 2:**
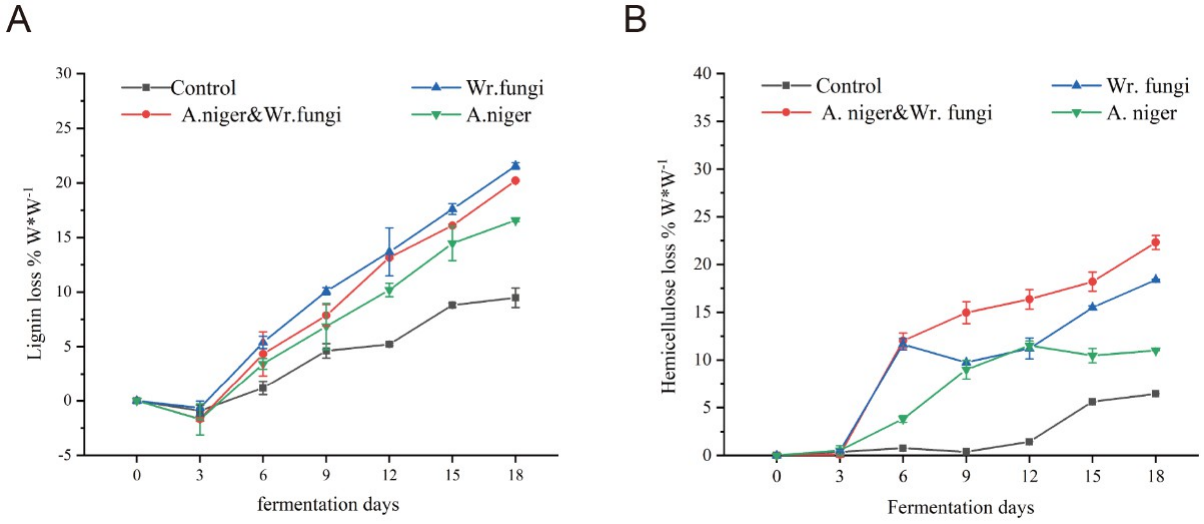
Loss ratios of lignin (A) and hemicellulose (B) of control bamboo shoots remains and bamboo shoots remains fermented with fungi.

To assess the selectivity of lignin and hemicellulose between different fungi, Fig 2 showed the loss rates of lignin and hemicellulose. The results showed that microbial fermentation caused the actual loss of lignin and hemicellulose, compared with the control. In the early stage of fermentation (0–3 days), the lignin content increased and the loss rate decreased. Lignin loss rate showed an increasing trend when the fermentation time was more than six days. Notably, *Wr*.*fungi* treatment exhibited the highest lignin loss rate (P<0.05) at 21.55%, emphasizing its superior selectivity for lignin degradation compared to *A*.*niger* and *A*.*niger&Wr*.*fungi*. This aligns with the high lignin loss rate observed in feed development by Yuqiong Wang et al. [14] using white rot fungi to ferment corn straw. Hemicellulose rates increased with fermentation time, with *A*.*niger&Wr*.*fungi*-treated BSR exhibiting the highest hemicellulose rate (P<0.05) at 22.32%.The increase of lignin content in the early stage of fermentation indicated that microorganisms would preferentially degrade saccharides such as starch and hemicellulose to produce H_2_O and CO_2_, resulting in the decrease of dry matter and the increase of lignin content [10]. However, the increased loss rate of hemicellulose indicated that hemicellulose was utilized at the early stage of fermentation. Previous studies have confirmed that hemicellulose can be used as an energy source for fungal growth for delignification, resulting in a continuous decrease in lignin and hemicellulose [32]. Studies have pointed out that white rot fungi have shown excellent results in degrading lignin because they secrete a large number of enzymes related to lignin degradation, such as LIP, Laccase, Filter paperase [11]. In addition, they are most effective for delignification due to a specific sequence of reactions by synergistic/cooperative actions of various lignolytic and cellulolytic enzymes [30]. However, the enzymes involved in hemicellulose degradation are glycoside hydrolases, endoglucanases, cellobiohydrolases I and II [33]. The enzymes that degrade lignin and hemicellulose are not the same, so the trends are not the same. In addition, the remaining cellulose after fungal treatment was more easily utilized by rumen microorganisms and enzymes than that before treatment [11].

### 3.3 Enzyme activity analysis

Table 1 delineates the impact of diverse treatments and fermentation durations on the resulting activities of cellulase, laccase, lipase (Lip), and filter paperase (FPase). Both culturing methods and fermentation duration wielded a significant influence on enzyme activities (P<0.05). In the intermediary fermentation period (3–12 days), BSR enzyme activity from fungal fermentation treatments surpassed that of the control group. Notably, the enzyme activity of treatment with *Wr fungi* reached its maximum on the sixth day of fermentation (Cellulase was 748.4 U/g; Laccase was 156.92 U/g; Lip was 291.61 U/g), and the highest values were higher than those of treatments. The increase of enzyme activity resulted in the decrease of ADF content (Table 2) and the loss of lignin content (Fig 2A). *A*.*niger* fermentation achieved its zenith enzyme activity on the 6th or 9th day (Cellulase: 606 U/g; Lip: 199.64 U/g; FPase: 0.286 U/g), albeit lower than the values observed for *Wr*.*fungi* and *A*.*niger&Wr*.*fungi*. Similarly, the loss rates of lignin and hemicellulose were lower compared to *Wr*.*fungi* or *A*.*niger&Wr*.*fungi* treatments (Fig 2).

**Table 1:** Enzyme activity of control bamboo shoot residues and bamboo shoot residues fermented with different fungi.

Item	Treatment	Sampling/day							SEM	P value		
		0	3	6	9	12	15	18		T	D	T*D
Cellulase	Control	347.5^Bd^	456.3^Bbc^	374.5^Cd^	454.6^Bc^	479.5^Cd^	521.3^Bab^	585.0^Aa^	94.16	<0.001	<0.001	<0.001
	*A*.*niger*&*Wr*.*fungi*	404.6^ABc^	477.2^Bbc^	612.0^Ba^	493.5^Bb^	463.3^Bbc^	459.6^Bbc^	435.8^Cbc^				
	*White-rot fungi*	383.9^Be^	451.8^Bd^	748.4^Aa^	613.8^Ab^	590.4^Abc^	605.4^Ab^	533.1^Bc^				
	*Aspergillus niger*	472.6^Ac^	584.8^Aa^	587.6^Ba^	606.1^Aa^	524.6^ABb^	454.0^Bc^	466.9^Cc^				
Laccase	Control	49.99^Cc^	51.46^Bc^	54.05^Cbc^	54.78^Cbc^	57.40^Cbc^	75.88^Bab^	91.78^Aa^	27.06	<0.001	<0.001	<0.001
	*A*.*niger*&*Wr*.*fungi*	72.56^Abc^	90.89^Abc^	98.60^Bbc^	101.90^Bab^	140.24^Aa^	84.08^ABbc^	60.64^Bc^				
	*White-rot fungi*	55.17^Cd^	78.35^Ac^	156.92^Aa^	129.94^Ab^	93.55^ABc^	98.39^Ac^	86.72^Ac^				
	*Aspergillus niger*	70.52^Ac^	89.64^Ab^	87.31^Bb^	99.74^Bab^	107.02^ABa^	61.13^Cc^	73.40^ABc^				
Lip	Control	105.65^b^	116.17^a^	96.73^Dcd^	100.53^Dbc^	81.13^Cf^	85.34^Ce^	92.63^Cde^	57.77	<0.001	<0.001	<0.001
	*A*.*niger*&*Wr*.*fungi*	103.81^f^	130.48^c^	131.70^Cc^	182.76^Ca^	176.17^Ba^	157.79^Bb^	116.71^Bd^				
	*White-rot fungi*	96.86^f^	153.79^e^	291.61^Aa^	263.90^Ab^	245.75^Ac^	228.46^Ad^	229.30^Ad^				
	*Aspergillus niger*	103.74^d^	157.76^bc^	199.64^Ba^	195.98^Ba^	172.77^Bab^	152.48^Bbc^	129.05^Bcd^				
Filter paperase	Control	0.155^Bd^	0.206^Cbc^	0.256^Aa^	0.236^Bab^	0.209^Bbc^	0.140^Cd^	0.174^Bcd^	0.055	<0.001	<0.001	<0.001
	*A*.*niger*&*Wr*.*fungi*	0.157^Bd^	0.334^Ba^	0.207^Bb^	0.186^Cb^	0.200^Bbc^	0.198^Bb^	0.167^Bcd^				
	*White-rot fungi*	0.222^Ac^	0.396^Aa^	0.258^Ab^	0.209^BCc^	0.277^Ab^	0.219^Bc^	0.160^Bd^				
	*Aspergillus niger*	0.212^Ac^	0.220^Cbc^	0.244^ABab^	0.286^Aa^	0.230^Bbc^	0.256^Aab^	0.239^Abc^				

Means values with different superscripts (a-d) in the same strains differ within sampling days (P < 0.05). Means values with different superscripts (A-D) in the same column differ within treatments (P < 0.05).

SEM, standard error of means.

**Table 2:** Chemical composition (g/kg DM) of control BSR and BSR fermented with fungi.

Item	Treatment	Sampling/day							SEM	P value		
		0	3	6	9	12	15	18		Treatment (T)	Day (D)	T*D
DM (g/100g)	Control	97.71^a^	95.50^ab^	98.07^Aa^	93.71^Bb^	98.30^Aa^	98.24^a^	98.50^Aa^	3.106	<0.001	<0.001	<0.001
	*A*.*niger*&*Wr*.*fungi*	96.12	93.17	97.72^A^	98.32^A^	95.20^B^	95.54	96.76^AB^				
	*White-rot fungi*	93.87	89.65	97.55^A^	98.03^A^	98.42^A^	98.37	98.06^AB^				
	*Aspergillus niger*	95.01^ab^	95.83^a^	89.71^Bb^	96.75^ABa^	97.33^ABa^	98.11^a^	95.88^Ba^				
CP	Control	11.84^b^	12.13^Bab^	12.22^Cab^	12.28^Bb^	12.34^Cb^	12.28^Cb^	12.22^Db^	1.609	<0.001	<0.001	<0.001
	*A*.*niger*&*Wr*.*fungi*	12.34^c^	12.59^ABbc^	13.50^Bb^	15.91^Aa^	16.19^Aa^	16.16^Aa^	16.09^Aa^				
	*White-rot fungi*	12.28	12.40^AB^	12.44^C^	12.66^B^	12.41^C^	12.31^C^	12.41^C^				
	*Aspergillus niger*	12.25^e^	12.78^Ad^	15.38^Abc^	16.09^Aa^	15.28^Bbc^	15.47^Bb^	14.91^Bc^				
Ash	Control	6.99	7.53	7.11^AB^	6.14^B^	8.30^B^	6.88^B^	6.65^C^	1.773	<0.001	<0.001	<0.001
	*A*.*niger*&*Wr*.*fungi*	7.81^bc^	6.98^c^	7.70^ABc^	10.15^Aab^	10.80^Aa^	10.90^Aa^	11.12^Aa^				
	*White-rot fungi*	7.88^ab^	6.66^c^	6.64^Bc^	8.83^ABa^	12.15^Bbc^	10.72^Bc^	10.26^Bab^				
	*Aspergillus niger*	7.57^c^	7.17^c^	9.73^Ab^	11.26^Aab^	15.28^Aa^	15.47^Aab^	14.91^Aab^				
ADF	Control	42.63^ABa^	41.60^Aab^	40.69^abc^	39.88^bc^	39.38^Abc^	39.80^Ac^	39.28^Ac^	1.874	<0.001	<0.001	<0.001
	*A*.*niger*&*Wr*.*fungi*	42.99^Aa^	41.51^Ab^	39.74^c^	38.83^cd^	38.84^Bde^	37.54^Bef^	37.07^Bf^				
	*White-rot fungi*	42.18^Ba^	40.94^Bb^	39.31^c^	37.69^d^	37.31^Cde^	36.64^Ce^	36.69^Ce^				
	*Aspergillus niger*	42.48^ABa^	40.85^Bb^	39.47^c^	39.05^c^	38.51^Bcd^	37.97^Bd^	37.64^Bd^				
NDF	Control	75.59^a^	74.43^Ab^	73.39^Ac^	72.72^Ac^	71.87Ad	70.90^Ae^	70.11^Af^	4.270	<0.001	<0.001	<0.001
	*A*.*niger*&*Wr*.*fungi*	73.26^a^	71.75A^Bb^	66. 37^Bc^	64.57^Bd^	63.66^Bd^	62.30^BCe^	60.58^Bf^				
	*White-rot fungi*	75.07^a^	73.66^Cab^	68.37^Ac^	67.37^Ad^	66.51^Ae^	64.43^ABf^	63.53^Bg^				
	*Aspergillus niger*	74.86^a^	73.06^Cb^	70.61^Bc^	68.52^Cd^	67.16^Be^	66.96^Ce^	66.42^Ae^				

Means values with different superscripts (a-d) in the same strains differ within sampling days (P < 0.05). Means values with different superscripts (A-D) in the same column differ within treatments (P < 0.05).

SEM, standard error of means.

Both cellulose and hemicellulose can be directly utilized by the ruminants. The lignin component in the cell wall hindered the utilization of polysaccharides by ruminants [34]. Fungi and bacteria can secrete a variety of lignin-degrading enzymes to degrade lignin, loosen molecular structure and improve rumen digestibility. These degrading enzymes mainly include Laccase, Lip, and FPase [35]. The enzyme activity of *Wr*. *fungi* fermentation surpassed that of *A*. *niger*, attributable to differences in carbon substrate. The decline in enzyme activity post-fermentation may stem from nutrient depletion in the substrate [36]. Previous studies substantiate that starch and pectin are initially utilized in the substrate, sparing lignin from degradation [32]. Cellulase, Laccase, Lip, and FPase in *A*.*niger* fermentation had lower enzyme activities in the early stage of fermentation. Accordingly, the loss rate of lignin and hemicellulose by *A niger* fermentation was also lower. However, the enzyme activity of *A*.*niger & Wr*.*fungi* treatment was lower than that of *Wr*.*fungi*, which may be due to the interaction between strains. Seungmin et al. [16] have shown that mixed fermentation is a very complex process, and pH and temperature will affect the properties of microorganisms. The decline in enzyme activity in later stages may be attributed to nutrient competition among strains. Our findings suggest that a fermentation duration of 4–10 days is optimal for most white rot fungi [37].

### 3.4 *In vitro* rumen fermentation

The effects of *Wr*.*fungi*, *A*.*niger* and *A*.*niger&Wr*.*fungi* fermented with BSR on NH3-N and pH in the fermentation products after 3 days of *in vitro* rumen fermentation are shown in Table 3. The results show that the treatment and duration had significant effect on NH_3_-N and pH. The strain used for fermentation and its duration had significant effects on NH_3_-N concentration and pH (P<0.05), the pH increased with prolonged duration. The NH_3_-N concentrations of *Wr*.*fungi* and *A*.*niger&Wr*.*fungi* fermented BSR were significantly increased compared with that of controls (P<0.05), the NH_3_-N concentrations of *Wr*.*fungi* fermentation increased with the extension of duration.

**Table 3:** *In vitro* fermentation products of control bamboo shoot residues and bamboo shoot residues fermented with different fungi.

Item	Treatment	Sampling/day							SEM	P value		
		0	3	6	9	12	15	18		Treatment (T)	Day (D)	T*D
pH	Control	5.65^b^	5.69^Bab^	5.72^Ca^	5.69^Cab^	5.73^Ba^	5.68^Dab^	5.73^Da^	0.706	<0.001	<0.001	<0.001
	*A*.*niger*&*Wr*.*fungi*	5.68^d^	5.77^Bd^	6.38^Bc^	7.15^Bb^	7.35^Aa^	7.43^Aa^	7.39^Ba^				
	*White-rot fungi*	5.67^b^	5.66^Bab^	5.72^Cab^	5.72^Cab^	5.73^Ba^	5.77^Ca^	5.77^Ca^				
	*Aspergillus niger*	5.65^e^	6.06^Ad^	6.94^Ac^	7.28^Ab^	7.33 ^Ab^	7.34^Bb^	7.47^Aa^				
NH_3_-N mmol/l	Control	32.44^Be^	34.25^Aa^	33.10^Cd^	33.51^Cc^	33.46^Cc^	33.76^Bb^	33.44^Cc^	5.925	<0.001	<0.001	<0.001
	*A*.*niger*&*Wr*.*fungi*	27.29^Dg^	31.75^Bf^	34.60^Bd^	46.04^Aa^	39.02^Bc^	33.38^Ce^	41.79^Bb^				
	*White-rot fungi*	33.44^Ae^	33.80^Ae^	35.11^Ad^	43.60^Bc^	47.30^Aa^	46.04^Ab^	47.34^Aa^				
	*Aspergillus niger*	30.06 ^Cc^	30.51^Cb^	28.86^De^	29.92 ^Dc^	31.93 ^Da^	29.39^Dd^	28.88 ^De^				

Means values with different superscripts (a-d) in the same strains differ within sampling days (P < 0.05). Means values with different superscripts (A-D) in the same column differ within treatments (P < 0.05).

SEM, standard error of means.

The concentration of NH_3_-N in *A*.*niger*&*Wr*.*fungi* treatment showed a downward trend in the late stage of fermentation. Furthermore, BSR was fermented by *A*.*niger* showed higher pH and lower NH_3_-N concentration. The pH of rumen is generally between 6.2 and 7.2. The change of pH will affect the population and abundance of rumen microorganisms and affect the absorption of nitrogen [38]. According to our data, the pH was lower in the early stage of fermentation, which was due to the fact that the rumen microorganisms were mostly Gram-negative bacteria, and the carbohydrate in the feed was fermented, resulting in a decrease in pH [39]. The NH_3_-N concentration can represent the balance between feed protein degradation and ammonia absorption of synthetic microbial proteins [40]. CP content increased in the early stage of fermentation and was positively correlated with NH3-N concentration. The increase of NH_3_-N concentration at the beginning of fermentation, promoted the utilization of nitrogen by ruminants and the synthesis of rumen microbial proteins [41].

### 3.5 Analysis of VFA content in *in vitro* rumen fermentation

The VFA content can provide about 70–80% energy source for ruminants, which is an important index to evaluate feed quality [11]. Therefore, we evaluated the total volatile fatty acids (TVFA) and the individual contents after rumen fermentation (Table 4). The content of TVFA in BSR fermented by fungi showed a downward trend compared with that of control, and decreased with the prolongation of fermentation time (P<0.05). Xueli et al. [11] elucidated that chitin, a substantial component of the fungal cell wall, posed resistance to rumen digestion, consequently reducing total VFA content. VFA is produced by carbohydrate fermentation, and about 95% of the TVFA produced by rumen fermentation are acetate, propionate, and butyrate [42].

**Table 4:** The content of VFAs *in vitro* fermentation of control bamboo shoot residues and bamboo shoot residues fermented with different fungi.

Item	Treatment	Sampling/day							SEM	P value		
		0	3	6	9	12	15	18		Treatment (T)	Day (D)	T*D
Total VFA g/L	Control	6.42^Ab^	4.97^Bf^	5.28^Ae^	5.23^Ae^	5.59^Ad^	7.12^Aa^	5.88^Ac^	1.430	<0.001	<0.001	<0.001
	*A*.*niger*&*Wr*.*fungi*	5.05^Cb^	5.29^Aa^	3.61^Cc^	3.10^Cd^	2.57^Ce^	2.39^Cf^	2.34^Df^				
	*White-rot fungi*	4.72^Dc^	4.47^Cd^	4.94^Bb^	4.74^Bc^	4.94^Bb^	3.37^Be^	5.57^Ba^				
	*Aspergillus niger*	5.59^Ba^	4.33^Db^	2.65^Dc^	2.28^Df^	2.35^De^	2.37^Cd^	2.36^Ce^				
Acetate %	Control	73.13^BCb^	73.07^Cb^	69.67^Be^	74.04^Aa^	72.85^Bb^	70.86^Bd^	72.36^Cc^	2.897	<0.001	<0.001	<0.001
	*A*.*niger*&*Wr*.*fungi*	72.51^Cb^	74.61^Aa^	68.02^Dc^	67.51^Cd^	66.64^Df^	66.06^Cg^	67.08^De^				
	*White-rot fungi*	73.66^ABa^	72.98^Cb^	72.93^Ab^	72.72^Bb^	71.40^Cc^	70.95^Bc^	73.89^Ba^				
	*Aspergillus niger*	74.04^Ac^	73.50^Bd^	68.32^Cf^	66.61^Dg^	73.11^Ae^	76.39^Aa^	75.25^Ab^				
Propionate %	Control	18.68^Bd^	18.98^Cc^	19.97^Cb^	18.58^Dd^	19.12^Cc^	20.48^Ba^	19.12^Cc^	1.083	<0.001	<0.001	<0.001
	*A*.*niger*&*Wr*.*fungi*	19.30^Ad^	17.88^De^	21.72^Aa^	19.75^Bb^	19.48^Bc^	19.39^Cdc^	19.34^Bdc^				
	*White-rot fungi*	18.39^Be^	19.76^Ac^	19.74^Cc^	19.57^Ccd^	20.14^Ab^	20.93^Aa^	19.42^Cd^				
	*Aspergillus niger*	19.15^Ac^	19.10^Bc^	20.68^Ba^	20.26^Ab^	18.13^Dd^	16.61^Df^	17.06^De^				
Butyrate %	Control	6.34^Acd^	5.79^Ae^	8.20^Aa^	5.28^Df^	6.01^Cde^	6.98^Bb^	6.59^Bbc^	1.745	<0.001	<0.001	<0.001
	*A*.*niger*&*Wr*.*fungi*	6.00^Abe^	5.40^Bf^	7.12^Bd^	9.09^Ab^	9.08^Ab^	9.56^Aa^	8.57^Ac^				
	*White-rot fungi*	5.65^Bb^	4.90^Cde^	5.11^Ccd^	5.45^Cbc^	6.24^Ba^	4.88^Cde^	4.63^Cf^				
	*Aspergillus niger*	4.81^Cc^	4.88^Cc^	6.90^Bb^	8.28^Ba^	4.14^Dd^	2.54^Df^	3.23^De^				
Valerate %	Control	0.50^a^	0.47^Cb^	0.55^Bb^	0.47^Bb^	0.49^Cb^	0.47^Db^	0.48^Cb^	0.254	<0.001	<0.001	<0.001
	*A*.*niger*&*Wr*.*fungi*	0.50^e^	0.51^Be^	0.93^Ad^	1.07^Abc^	1.18^Aa^	1.11^Ab^	1.03^Ac^				
	*White-rot fungi*	0.52^b^	0.48^Cbc^	0.51^Bbc^	0.47^Bc^	0.49^Cbc^	0.60^Ca^	0.47^Cc^				
	*Aspergillus niger*	0.48^c^	0.56^Ac^	0.89^Ab^	1.11^Aa^	1.00^Bab^	0.87^Bb^	0.86^Bb^				
A/P	Control	3.92^Bb^	3.85^Bc^	3.49^Bf^	3.99^Aa^	3.81^Cd^	3.46^Bg^	3.78^Be^	0.335	<0.001	<0.001	<0.001
	*A*.*niger*&*Wr*.*fungi*	3.76^Cc^	4.17^Aa^	3.13^Df^	3.42^Ce^	3.98^Bb^	3.41^Ce^	3.47^Cd^				
	*White-rot fungi*	4.01^Aa^	3.69^Cc^	3.69^Ac^	3.72^Bbc^	3.55^Dd^	3.39^Ce^	3.81^Bb^				
	*Aspergillus niger*	3.87^Bb^	3.85^Bd^	3.30^Ce^	3.29^De^	4.03^Ac^	4.60^Aa^	4.41^Ab^				

Means values with different superscripts (a-d) in the same strains differ within sampling days (P < 0.05). Means values with different superscripts (A-D) in the same column differ within treatments (P < 0.05).

SEM, standard error of means. A/P, Acetate/ Propionate.

VFA content serves as a crucial indicator of feed nutritional value and rumen fermentation status, profoundly influenced by NDF content [43]. Variations in VFA content among treatments stem from their disparate NDF compositions. Lower NDF content was found to favor the production of propionate and butyrate, as confirmed by our data. Acetate, primarily generated through the microbial action on cellulose and hemicellulose [44], was impacted by the distinct NDF content in our treatments. A.niger&Wr.fungi treatment, boasting the lowest NDF content and the highest hemicellulose loss rate, limited acetate synthesis. Acetate amount accounts for the largest proportion and is the substrate for the rumen to provide energy sources. *A*.*niger&Wr*.*fungi* fermentation had the lowest acetate/propionate (A/P) ratio compared with other fermentation methods after fermentation, and the proportion of propionate reached the maximum (21.72%) on the sixth day of fermentation. Studies had confirmed that the proportion of refined grains increases, and the rumen tends to propionate fermentation, on the contrary, to acetate fermentation [45]. Propionate was a precursor substance for the synthesis of glucose, which provided more energy for ruminants [46], *A*.*niger&Wr*.*fungi* fermentation produced higher proportion of propionic acid in favor of rumen utilization of feed. Approximately 90% of butyrate in rumen epithelial cells was converted into beta-hydroxybutyrate and acetoacetate, which promoted the development of rumen metabolic function [47]. In this study, *A*.*niger&Wr*.*fungi* fermentation had the highest proportion of butyrate compared with other treatments, which promoted the metabolic function of rumen and made up for the shortcomings of fermentation alone. And the results showed that fermentation for 6 days could help rumen digestion.

## 4. Conclusions

*A*.*niger* and *Wr*.*fungi* and their co-culture (*A*.*niger*&*Wr*.*fungi*) were used to ferment BSR to produce ruminant feed. The fermented BSR significantly increased the protein content, decreased the NDF and ADF content, and caused the loss of lignin and hemicellulose. The protein content of *A*.*niger*&*Wr*.*fungi* fermentation was the highest, *Wr*.*fungi* fermentation had the best ability to degrade lignin, and treatment with *A*.*niger*&*Wr*.*fungi* had the best ability to degrade hemicellulose. The NH_3_-N concentrations of *Wr*.*fungi* and *A*.*niger&Wr*.*fungi* fermentation were significantly increased. The lowest acetate/propionate was found in *A*.*niger*&*Wr*.*fungi* fermentation, which increased rumen digestibility. Fungal co-culture made up for the shortcomings of monoculture by improving protein content, degrading NDF and optimizing VFA ratio. The optimal duration for fermentation is 6 days, taking into account all factors. Our work provided a theoretical basis for promoting the development of fermented ruminant feed in future.

## Supporting information

S1 Raw data (ZIP)
