# Supplementary figures and images for: Enhancing quality of ruminant feed through fungal treatment: Usage of bamboo shoot residues
Source: PLoS One. 2024 May 28;19(5):e0302185. doi: 10.1371/journal.pone.0302185 (PMC11132483; doi:10.1371/journal.pone.0302185)

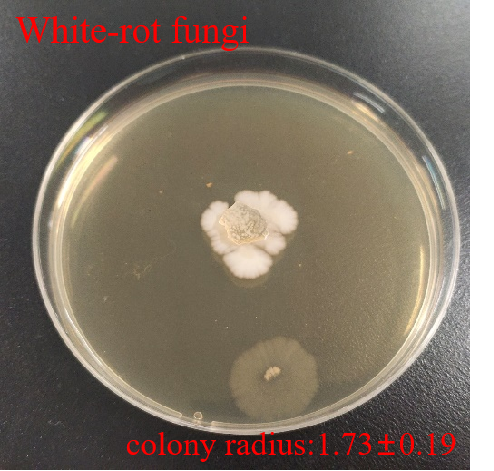

Supplement: S1 Raw data — (ZIP) [file pone.0302185.s001.zip › Experimental raw data/░╫╕»╛·.png]

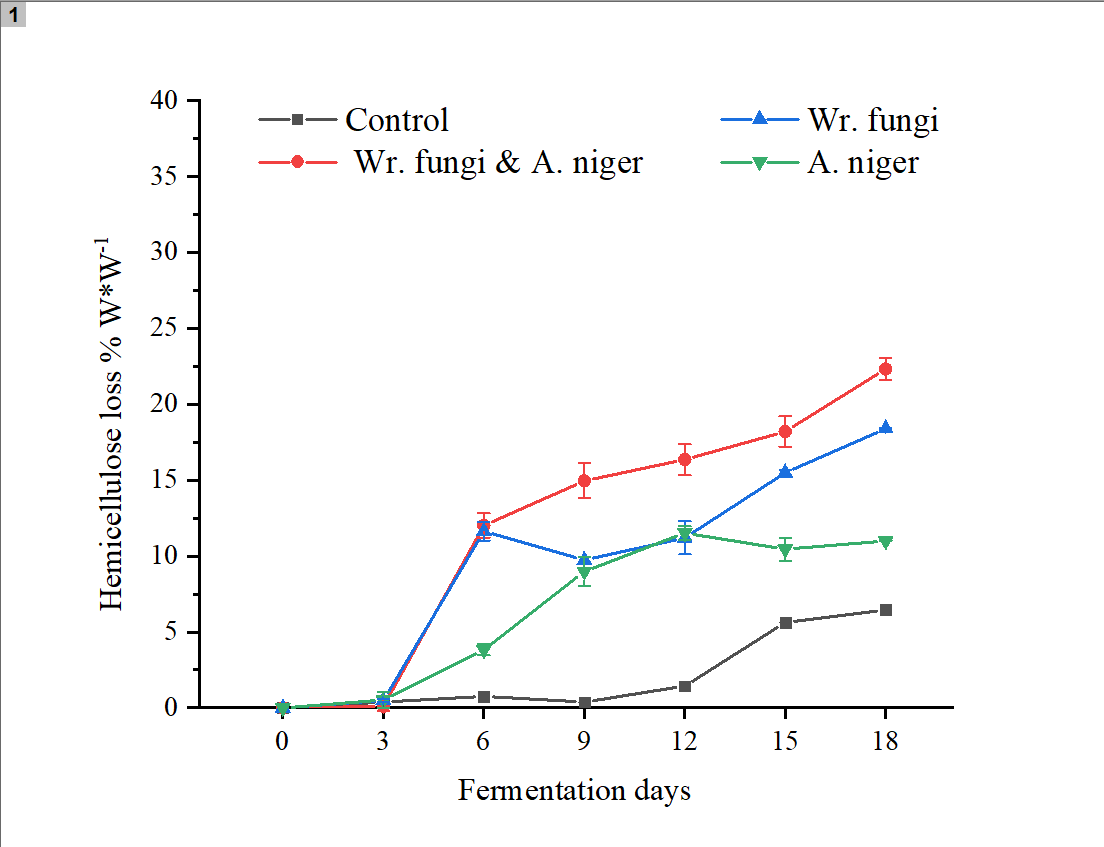

Supplement: S1 Raw data — (ZIP) [file pone.0302185.s001.zip › Experimental raw data/░δ╧╦╬1⁄4╦╪╦≡╩o┬╩..png]

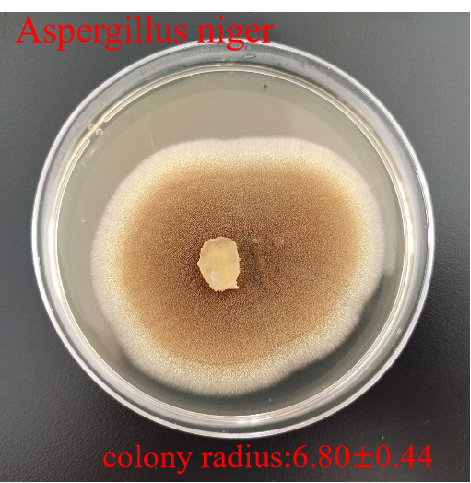

Supplement: S1 Raw data — (ZIP) [file pone.0302185.s001.zip › Experimental raw data/║┌╟·├╣.png]

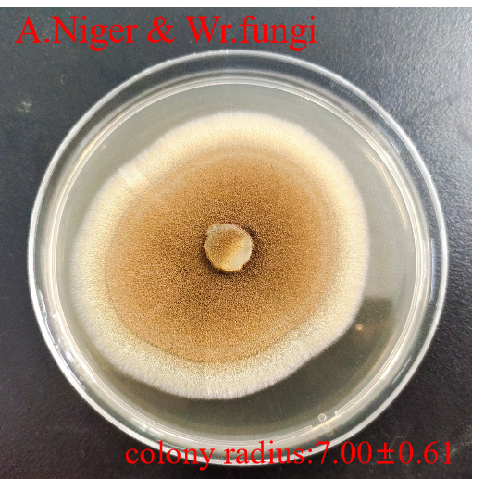

Supplement: S1 Raw data — (ZIP) [file pone.0302185.s001.zip › Experimental raw data/╗∞║╧.png]
